# The Molecular Properties and Roles of *Pannier* in *Harmonia axyridis*’s Metamorphosis and Melanin Synthesis

**DOI:** 10.3389/fphys.2022.909258

**Published:** 2022-05-03

**Authors:** Renbin Tian, Xu Chen, Mengmeng Wu, Qingxuan Xu, Su Wang, Liansheng Zang, Da Xiao

**Affiliations:** ^1^ Jilin Engineering Research Center of Resource Insects Industrialization, Institute of Biological Control, Jilin Agricultural University, Changchun, China; ^2^ Institute of Plant Protection, Beijing Academy of Agriculture and Forestry Sciences, Beijing, China; ^3^ Key Laboratory of Green Pesticide and Agricultural Bioengineering of Ministry of Education, Guizhou University, Guiyang, China

**Keywords:** pannier, melanin, development, *Harmonia axyridis*, metamorphosis

## Abstract

The GATA transcription factor *Pannier* is identified as the major regulatory gene in color pattern formation in the Asian multi-colored ladybird beetle (*Harmonia axyridis*). however, the mechanisms of *Pannier* in regulating melanin synthesis and development in *H. axyridis* remain elusive. In this study, we identified and characterized *Pannier* in *H. axyridis* (*HaPnr*) and showed it to have two alternative spliced variants named *HaPnr-α* and *HaPnr-β*. Analyses of developmental stage expression patterns revealed that *HaPnr*, *HaPnr-α* and *HaPnr-β* were constitutively expressed throughout all developmental stages. To examine the role of *HaPnr* in *H. axyridis* development, RNA interference was performed in late larvae (the fourth instar) and early pupae (the first day of pupa stage). The transcript levels of *HaPnr* were effectively suppressed after the injection of double-stranded RNA of *HaPnr* (ds*HaPnr*). The fourth instar larvae injected with ds*HaPnr* reduced the pupation rates to only 61.50%, compared with 88.5% in the ds*GFP*-injected group. The un-pupated larvae gradually died after 1 week, and visually unaffected pupae emerged into abnormal adults with malformed hind wings and melanin absent from the cuticle. These abnormal adults gradually died 10 days after eclosion. However, when early pupae were injected with ds*HaPnr*, the normal eclosion rate was achieved at 88.41% on day 6 after the injection. In addition, these successful eclosion adults also showed an absence of melanin in the cuticle, but they could mate normally and have normal fecundity as compared with the control. We further demonstrated that the suppression of *HaPnr-α* or *HaPnr-β* individually did not affect the pupation and eclosion process. The suppression of *HaPnr-α* expression resulted in elytra melanin decreasing in both the *conspicua* and the *succinea* subgroup in *H. axyridis*. Even though the suppression of *HaPnr-β* expression only affected the melanin synthesis in the *succinea* subgroup, it significantly prolonged the time taken for melanin synthesis to occur in the *conspicua* subgroup in *H. axyridis*. These results indicate that *HaPnr* plays an essential role in insect development, especially during their metamorphosis, and also support our hypothesis that *HaPnr* could regulate melanin synthesis in *H. axyridis* under the combined action with its two splicing variants, *HaPnr-α* and *HaPnr-β.*

## Introduction

Insects are among the species with the most abundant phenotypic diversity in the world (Moczek, 2010). Phenotypic diversity occurs with important biological and physiological significance in insect development and evolution (Chau and Goodisman, 2017). Phenotypic plasticity is a regulatory mechanism for organisms to respond to environmental changes. In insects, melanization is a significant and common phenomenon in phenotypic diversity on the cuticle. Since the first finding of a melanized individual of *Bistom betularia* in Manchester in 1864, many scholars have focused on and examined the melanization phenomenon, its creation mechanism, and its evolutionary significance (True, 2003). Many studies have demonstrated that melanization occurs with important physiological meaning in insects, such as melanic individuals protecting themselves through mimicry, such as the swallowtail butterfly and *Papilio glaucus* in Lepidoptera (Koch et al., 2000), while melanic individuals of *Oncopeltus fasciatus* have higher fitness in low temperature (Sharma et al., 2016). In addition, the intraspecific diversification of elytra melanin patterns in ladybird beetle showed obvious mating selection preference (Wang et al., 2009; Wang et al., 2013). As a result, a foundation for developing particular melanized strains needs to be established by studying the melanization mechanism in insects. Previous studies have demonstrated that there are significant differences in the melanization mechanism between insects and vertebrates because of pigment synthesis differences. Vertebrates use specialized cell types and cell migration to create body pigment patterns (Hoekstra, 2006), while insects typically produce their body color pigment (or precursor) in epidermal cells then secrete these substances into the developing cuticle (True, 2003). The synthesis and deposition of insect melanin pigments can be influenced by environmental factors and be regulated by pivotal genes (Wittkopp and Beldade, 2009; Van der Burg et al., 2020).

The Asian multi-colored ladybird beetle, *Harmonia axyridis* (Coleoptera: Coccinellidae), is known for its voracious predatory capacity on various key pests (Koch, 2003; Chen et al., 2019a). In addition, it also works as an ideal model organism to study insect phenotype variations because of more than 200 different elytral color patterns in *H. axyridis* (Gautier et al., 2018; Ando and Niimi, 2019). There are four common color patterns of elytra that have been widely studied in *H. axyridis*, which include: *succinea*, *axyridis*, *spectabilis*, and *conspicua* (Ando et al., 2018; Gautier et al., 2018; Xiao et al., 2020). This striking intraspecific variation has prompted the investigation of its genetic, biochemical, and evolutionary meaning (Bezzerides et al., 2007; Ando et al., 2018). A previous study demonstrated that the elytra color pattern variation in *H. axyridis* is a melanization phenomenon in the cuticle (True, 2003). In our previous studies, we have confirmed that dopamine melanin synthesis is the primary pathway in the elytra of *H. axyridis* (Chen et al., 2019b; Xiao et al., 2020). Meanwhile, the aspartate-*β*-alanine-NBAD pathway being able to regulate the number and size of melanin spots in elytra has been reported in *H. axyridis* (Zhang et al., 2020; Zhang et al., 2021). Recently, new insights into the effect of the transcription factor *Pannier* on the regulation of pigment synthesis were discovered in *H. axyridis* (Niimi and Ando, 2021). With the advancement of next-generation sequencing (NGS) technologies, the ortholog of the *Drosophila melanogaster Pannier* gene was identified as the regulatory gene of wing color pattern polymorphism at the *h* locus in *H. axyridis*. Duals regulate functions of the *Pannier* gene on color pattern polymorphism in *H. axyridis*. First, repeated inversions at the upstream region of the first intron of *Pannier* resulted in the four common subgroup formations from the evolutionary genetic aspect. Second, *Pannier* could regulate melanin synthesis, further influencing color pattern formation from the physiological aspect (Ando et al., 2018; Gautier et al., 2018).

The biological function of *Pannier* has been studied in the genetically tractable organism *D. melanogaster*. *Pannier* was first reported in *D. melanogaster* in 1993 (Ramain et al., 1993), and is one of five GATA transcription factors in insects (Minakhina et al., 2011). Previous studies demonstrated that *Pannier* plays important roles in several developmental progresses in *D. melanogaster*. In the embryo’s development, *Pannier* is essential for dorsal closure (Herranze and Morata, 2001) and hemocyte maturation and differentiation (Minakhina et al., 2011). During imaginal development, the function of *Pannier* is to control imaginal disc development, while it acts upstream of *wingless* (*wg*) to direct dorsal eye disc development (Maurel-Zaffran and Treisman, 2000). Otherwise, *Pannier* controls compound eye size by activating target genes during dorsal–ventral axis formation in eye–antennal disc development (Buchberger et al., 2021). Moreover, *Pannier* also plays an important role in establishing and maintaining proper heart function in *D. melanogaster* (Gajewski et al., 1999; Qian and Bodmer, 2009; Lovato et al., 2015). However, the detailed function of *Pannier* in *H. axyridis* is still unknown. Thus, it is necessary to investigate the physiological function of *Pannier* in *H. axyridis* to deepen the understanding of the role of *Pannier* in the development of insects.

Even though the role of *Pannier* in elytral pigmentation during pupal development was discovered in *H. axyridis*, the detailed regulatory mechanisms remain elusive. To better understand the regulation mechanisms of *Pannier* in *H. axyridis* and further its physiological function in development, we: 1) sequenced and characterized cDNA, putatively encoding *Pannier* from *H. axyrids*, an emerging model organism in multiple phenotype evolution studies; 2) examined the developmental stage-dependent expression profiles of *HaPnr*; and 3) investigated the roles of *HaPnr* in melanin synthesis and insect development by using RNA interference. Our results provide crucial evidence that *Pannier* regulates melanin synthesis through a special action mode with its splicing variants and is an essential gene during the developmental process in *H. axyridis.*

## Materials and Methods

### Insect Culture

Adults of *H. axyridis* were collected from wheat fields (39°95′ N, 116°28′ E) on the experimental field of the Beijing Academy of Agriculture and Forestry Sciences (BAAFS), Beijing, China, in May 2014. The ladybird beetles were transported to an automated environmental management system controlled (Sunauto, Beijing, China) rearing room. They were maintained in aluminum frame cages (50 cm × 50 cm × 50 cm) with 100-mesh plastic gauze coverings at 25 ± 1°C, 60% relative humidity, and 16 h: 8 h (Light: Dark) photoperiod. Each cage housed 40 pairs of adults, which were fed daily on vicia aphids, *Megoura japonica* Matsumura (Hemiptera: Aphididae) on seedlings of broad bean, *Vicia faba* L., cv. ‘LinCan-5’).

### Total RNA Isolation and Reverse Transcription

TRIzol reagent (Invitrogen, Carlsbad, CA, United States) was used to extract total RNA from pupa of *H. axyridis* samples, and the RNA concentration was determined at 260 nm using a NanoDrop ONE spectrophotometer (Thermo Fisher Scientific, Waltham, MA, United States). After the total RNA (1.0 μg) was used for cDNA synthesis using the First Strand cDNA Synthesis Kit (Vazyme, Nanjing, China) with oligo (dT)_18_ as the primer in a 20 μL reaction system. The first-strand cDNA was used in all subsequent analyses.

### Subcloning and Sequencing of *HaPnr* Coding Sequences

One pair of gene specific primers were designed on the *HaPnr* gene prediction in NCBI (Accession number: LC269051.1) to amplify open reading frame (ORF) fragments by PCR for the full-length cDNA corresponding to the entire protein coding regions (Table 1). The PCR products were subjected to electrophoresis on 1% agarose gel. The PCR bands were excised and purified using QIAEX II Agarose Gel Extraction Kit (QIAGEN, Hilden, Germany). The purified PCR fragment was ligated into pcDNA3.1 (+) vector (Invitrogen, Carlsbad, CA, United States). The ligation mixtures were then used to transform Fast-T1 bacterial cells (Vazyme, Nanjing, China). Plasmids were isolated from the bacterial cells and sequenced by a DNA sequencing company (Sangon, Shanghai, China).

**TABLE 1:** Primers used to amplify *HaPnr* cDNA sequence, synthesize dsRNA and analyze transcript levels.

Primer name	Sequence (5′-3′)	Tm (°C)	Product Size (bp)
*PCR for cDNA sequence*			
*HaPnr*-F	ATGTTCCACACCGGCGCC	64.4	1083
*HaPnr*-R	TTA​TGT​CGT​AGC​CAT​CAG​TTT​GGC	63.4	
*PCR for dsRNA synthesis*			
ds*GFP*-F	taa​tac​gac​tca​cta​tag​gga​gaC​AGT​GCT​TCA​GCC​GCT​AC	69.7	305
ds*GFP*-R	taa​tac​gac​tca​cta​tag​gga​gaG​TTC​ACC​TTG​ATG​CCG​TTC	68.8	
ds*HaPnr-*F	taa​tac​gac​tca​cta​tag​ggG​GAC​TGT​GCT​GCA​CGA​ACT	67.1	303
ds*HaPnr-*R	taa​tac​gac​tca​cta​tag​ggG​GGA​TGG​TTT​TGA​GAT​GAC​G	65.9	
ds*HaPnr-* *α-*F	taa​tac​gac​tca​cta​tag​ggA​GTG​CCA​TGG​AGT​TCC​AGT​T	59.6	94
ds*HaPnr-* *α-*R	taa​tac​gac​tca​cta​tag​ggA​GTG​CCC​TGT​ACC​ATC​TCT​CC	59.5	
ds*HaPnr-* *β-*F	taa​tac​gac​tca​cta​tag​ggT​ACG​AAA​GTA​ACC​CGT​ACC​CC	60.1	60
ds*HaPnr-* *β-*R	taa​tac​gac​tca​cta​tag​ggT​CGA​TAT​GGC​GCT​AGA​TTC​C	60.1	
*Reverse transcription quantitative PCR* (*RT-qPCR*)			
*HaPnr(Q)-*F	TAC​CCA​GAC​CTT​GGA​ACG​AC	60.0	135
*HaPnr(Q)-*R	CCG​AAG​ATT​TGC​TGG​TAA​GG	59.7	
*HaPnr-* *α*(*Q*)*-*F	TTG​TGT​AAC​GCT​TGT​GGT​TTG	59.7	100
*HaPnr-* *α*(*Q*)*-*R	ACA​GTC​CCA​AGC​GTC​TGG​T	60.7	
*HaPnr-* *β*(*Q*)*-*F	CTA​GCG​CCA​TAT​CGA​AAA​CC	59.7	211
*HaPnr-* *β*(*Q*)*-*R	TCC​TCT​TAC​GGG​TCT​GGA​TG	60.1	
*Harp49*-F	GCC​GTT​TCA​AGG​GAC​AGT​AT	56.7	84
*Harp49*-R	TGA​ATC​CAG​TAG​GAA​GCA​TGT​G	57.8	

## Analyses of *HaPnr* Coding Sequences, Deduced Amino Acid, and Gene Sequences

The amino acid sequence of a putative HaPnr protein was deduced from its cDNA, and molecular mass and isoelectric point of the deduced protein were calculated by using online tools of ExPASy website (http://www.expasy.org/tools/). Structure homology modeling was done using SWISS-MODEL (https://swissmodel.expasy.org). Multiple amino acid sequence alignment of all known insect Pnrs found in UniProt was carried out using ClustalW (http://www.ebi.ac.uk/Tools/msa/clustalw2/). We performed insect *Pannier* phylogenetic analysis using the neighbor-joining algorithm by using Mega X software. To evaluate the branch strength of the tree, a bootstrap analysis of 1000 replications was performed. The gene structure of *HaPnr* was revealed by comparing the full-length cDNA sequence with its corresponding gene sequence from genome data (http://v2.insect-genome.com/Organism/418).

### Analysis of Expression Profiles by Reverse Transcription Quantitative PCR

The relative transcript levels of *HaPnr* including its two splicing variants, *HaPnr-α* and *HaPnr-β*, were analyzed by reverse transcription quantitative PCR (RT-qPCR) using SYBR Green with the Applied Biosystems^®^ Real-time PCR Instrument (ABI Laboratories, Hercules, CA, United States). For developmental expression profiling, samples were prepared from the embryos of one-, two- and three-day-old eggs, larvae of all four instars, pupae ranging from one to 4 days in age, and initial eclosion adults, including males and females. Gene-specific primers (Table 1) were designed using the Primer3Plus (Untergasser et al., 2007) based on our obtained *HaPnr, HaPnr-α* and *HaPnr-β* sequence information. The ribosomal protein S49 (*Harp49*) in *H. axyridis* was used as an internal reference gene.

The optimized quantitative PCR program consisted of an initial denaturation at 95°C for 30 s, followed by 40 cycles of 95°C for 10 s, and 60°C for 30 s. After PCR, amplification specificity was verified by obtaining the dissociation curve, in which the PCR product were followed by one repeat of 95°C for 15 s, 60°C for 1 minute, and 95°C for 15 s. The specificity of each reaction was evaluated based on the melting temperatures of the PCR products. RT-qPCR was performed with three biological replicates, each with three technical replicates. The transcript levels of *HaPnr*, *HaPnr-α* and *HaPnr-β* were expressed as the normalized transcript abundance using *Harp49* as an internal reference gene, individually. The relative *HaPnr*, *HaPnr-α* and *HaPnr-β* transcript levels were calculated according to the 2^-△△Ct^ method.

### Functional Analysis of *HaPnr*

The role of *HaPnr* in *H. axyridis* development was investigated using RNA interference (RNAi). Double stranded RNAs (dsRNAs) were synthesized using MEGAscript^®^ RNAi Kit (Invitrogen, Carlsbad, CA, United States) according to the manufacturer’s instructions. Relevant information on the primers used for dsRNA synthesis is given in Table 1. The fourth instar larvae and one-day-old pupae were chosen for injections of *HaPnr*, *HaPnr-α* and *HaPnr-β* dsRNA (ds*HaPnr,* ds*HaPnr-α* and ds*HaPnr-β*) at doses of 300 ng/individual; To serve as controls, similar numbers of the same life stages were injected with dsRNA of the green fluorescent protein gene (ds*GFP*) at the same dose. The injection of ds*GFP* resulted in a mortality rate of less than 10%. Each RNAi experiment was carried out three times with at least 40 insects each time. The injected insects were reared in regular conditions, and phenotypes (elytral coloration) of the insects was recorded daily following injection. RT-qPCR was used to monitor the change in *HaPnr, HaPnr-α* and *HaPnr-β* transcript level after the injection. Three insects from each time point were then pooled as a sample for total RNA extraction. The analysis was performed with three biological samples per time point, with three technical replicates per sample. Phenotype images were taken using Zeiss Microscope SteREO Discovery V20 (Carl Zeiss, Germany), all using the same magnification, exposure time, and light intensity. Images were then selected for depiction of the most representative phenotypes.

To examine the effect of *HaPnr* suppression on fecundity in *H. axyridis*, the one-day-old pupae were injected with ds*HaPnr* (300 ng/pupa) or ds*GFP* as controls. After the injected pupae developed into adults, the males and females were separated and paired randomly due to the disappeared characteristic of color type in adults when *HaPnr* was suppressed in the pupa stage. Each treatment consisted of three pairs of a male and a female, and each treatment was repeated three times. Eggs were collected for 15 days after eclosion. Egg hatchability was examined 3 days after the eggs were collected.

### Statistical Analysis

The level of *HaPnr*, *HaPnr-α* and *HaPnr-β* transcript in RT-qPCR analysis was expressed as a percentage of the level in the controls by dividing the relative expression value (REV) in the ds*HaPnr*-injected insects by REV in the ds*GFP*-injected insects and multiplying by 100. The REVs from developmental stages were arcsine square-root transformed before being subjected to ANOVA followed by Duncan’s test. Percent data from the RNAi experiments were subjected to ANOVA followed by Duncan’s test to separate means (IBM SPSS Statistics 22.0 software).

## Results

### Analysis of *HaPnr* Gene Sequences, cDNA and Amino Acid

**FIGURE 1:**
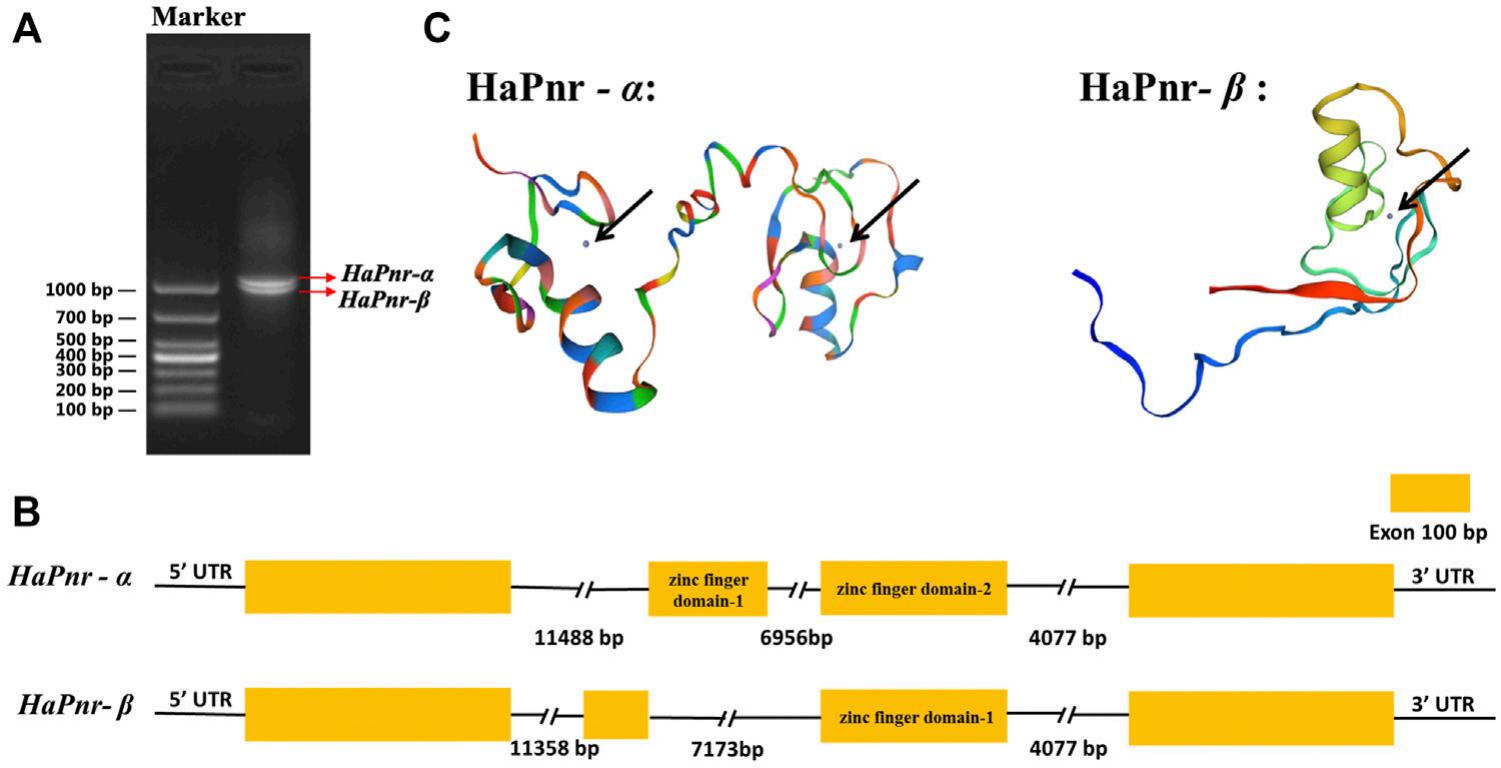
Electropherogram of *HaPnr* gene amplified, DL1000 (Takara Bio Inc., Japan) was used as DNA marker for agarose gel electrophoresis **(A)**. Schematic diagram for the organization of *HaPnr-α* and *HaPnr-β* **(B)** and the location of zinc-finger domain in the predicted protein structure chart of *HaPnr-α* and *HaPnr-β* (black arrow) **(C)**.

The length of the *HaPnr* gene sequence is 23,604 base pairs (bp) from the transcription start site to the transcription end site, containing four exons and three introns. The *HaPnr* gene is located on chromosome 4. *HaPnr* has two splicing variants, *HaPnr-α* and *HaPnr-β* (Figure 1A). Sequencing analysis of the components of *HaPnr-α* and *HaPnr-β* found that alternative splicing occurred in second exon (Figure 1B). The open read frame (ORF) of *HaPnr-α* is 1083 bp, encoding 360 amino acids (aa). The predicted molecular mass and isoelectric point are 38.91 kDa and 9.19, respectively. The ORF of *HaPnr-β* is 996 bp, encoding 331 aa. The predicted molecular mass and isoelectric point are 35.96 kDa and 9.30, respectively. The zinc-finger domain is the representative characteristic of the transcription factor *Pannier*. Our results show that *HaPnr-α* has two zinc finger domains and *HaPnr-β* has one zinc finger domain based on the SWISS-MODEL analysis (Figure 1C). Detailed information regarding the zinc-finger domain located on *HaPnr* sequences is shown in Supplementary Figures S1, S2.

### Phylogenetic Relationship of *HaPnr* With Other Insect Panniers

All amino acid sequences of Pannier for phylogenetic analysis were obtained from the UniProt database, forming five distinctive groups representing different orders: Coleoptera, including *Coccinella septempunctata* (*CsPnr*, amino acid sequence identity to *HaPnr-α* 92.47%, to *HaPnr-β* 76.15%), *Tribolium castaneum* (*TcPnr*, identity to *HaPnr-α* 78.80%, to *HaPnr-β* 70.31%), *Onthophagus binodis* (*ObPnr*, identity to *HaPnr-α* 72.73%, to *HaPnr-β* 59.62%), *O. sagittarius* (*OsPnr*, identity to *HaPnr-α* 72.73%, to *HaPnr-β* 60.09%); Diptera, including *D. quadrilineata* (*DqPnr*, identity to *HaPnr-α* 56.10%, to *HaPnr-β* 42.70%), *D. melanogaster* (*DmPnr*, identity to *HaPnr-α* 61.11%, to *HaPnr-β* 51.35%), *Anopheles gambiae* (*AgPnr*, identity to *HaPnr-α* 59.25%, to *HaPnr-β* 40.58%), *Megaselia abdita* (*MaPnr*, identity to *HaPnr-α* 66.33%, to *HaPnr-β* 48.78%), *Calliphora vicina* (*CvPnr*, identity to *HaPnr-α* 57.88%, to *HaPnr-β* 39.47%), *Ceratitis capitata* (*CcPnr*, identity to *HaPnr-α* 58.08%, to *HaPnr-β* 47.33%), *Clogmia albipunctata* (*CaPnr*, identity to *HaPnr-α* 75%, to *HaPnr-β* 40.62%); Hemiptera, including *Oncopeltus fasciatus* (*OfPnr*, identity to *HaPnr-α* 81.61%, to *HaPnr-β* 72.84%); Lepidoptera, including *Danaus plexippus* (*DpPnr*, identity to *HaPnr-α* 50.42%, to *HaPnr-β* 39.66%) and Lxodida, including *Ornithodoros erraticus* (*OePnr*, identity to *HaPnr-α* 44.68%, to *HaPnr-β* 37.53%). Based on the amino acid identity levels, *HaPnr* appears to be more related to those in Coleoptera than to those in other orders.

### Developmental Stage-specific Expression Patterns of *HaPnr*

Analyses of the developmental stage-specific expression pattern of *HaPnr* by using RT-qPCR in the egg, larval, pupal, and adult stages showed constitutive expression (Figure 3A). The highest expression occurred in one-day-old eggs, and other expression peaks were found in the first day of larva and pupa stage. Based on the two splicing variants of *HaPnr*, two specifics primers of RT-qPCR were designed on the specific domain to analyze developmental stage expression patterns of *HaPnr-α* and *HaPnr-β*, respectively (Supplementary Figure S3). Our results show that these two splicing variants have similar expression tendency with *HaPnr.* Additionally, the highest expression also occurred in one-day-old eggs (Figure 3B,C).

### RNAi of *HaPnr* in the Fourth Instar Larvae and Its Effect on Pupation, Eclosion and Elytral Melanization

Due to two splicing variants having occurred in *HaPnr*, the dsRNA corresponding to *HaPnr* was designed on the common region and the primers of RT-qPCR were also designed on the common region that has no overlap with dsRNA domain (Supplementary Figure S3). When the fourth instar larvae of *H. axyridis* were injected with ds*HaPnr*, the transcript levels of *HaPnr* were significantly suppressed on days 4, 6 and 8 after injection; the remaining *HaPnr* was only 40% that of the ds*GFP*-injected group on day 4 after injection (Figure 4A). Consequently, the injection of ds*HaPnr* in the fourth instar larvae led to steadily decreasing survival frequency. By day 16 after the injection, only a 16.4% survival ratio was observed in ds*HaPnr*-injected larvae (Figure 4B). When *HaPnr* was suppressed in the fourth instar larvae, a significantly decreased pupation rate was observed as compared with the control (Figure 4C). In the control group with ds*GFP* injection, 88.5% of larvae could successfully pupate. However, in the ds*HaPnr*-injected group, approximately 61.5% of larvae could pupate, but these pupae showed an abnormal phenotype in which melanin on the cuticle was decreased as compared with the control group. The remaining 38.5% of larvae in the ds*HaPnr*-injected group could not accomplish the pupate process and died in larva form. Furthermore, these dead individuals showed epidermis softening and an abnormal darkening in color (Figure 4E). Suppressing the expression level of *HaPnr* in the fourth instar larvae also affects the eclosion process. These successfully pupating individuals with abnormal melanization in the epidermis were examined to calculate the eclosion rate. However, 100% of insects with an overall abnormal eclosion in the ds*HaPnr* injected group showed a malformed hind wing and the exuvium was attached to the body. In addition, the melanin in the head, pronotum and elytra were totally absent as compared with the control. We also observed that the original melanin synthesis areas are transformed into a yellow color, leading to a loss of color pattern characteristic in the elytra (Figure 4F). All the abnormal adults died within 10 days after the eclosion.

### RNAi of *HaPnr* in One-Day-Old Pupae and Its Effect on Eclosion and Elytral Melanization

When early (1 day old) pupae were injected with ds*HaPnr*, the transcript levels of *HaPnr* were also significantly suppressed on days 2 and 4 after the injection. The *HaPnr* transcript levels were suppressed by 44.5% on day 2 after the injection of ds*HaPnr* (Figure 5A). Unlike with ds*HaPnr* injection in the fourth instar larvae, the suppressed expression level of *HaPnr* in one-day-old pupae did not affect the metamorphosis process from pupae to the adult stage (Figure 5C). Even though the pupae could eclose, but these adults showed an abnormal phenotype in which melanin was missing from the head, pronotum and elytra in *H. axyridis*. As with the phenotype in which *HaPnr* was suppressed in the fourth instar larvae, this melanin-lacking adult also displayed an original melanin synthesis area which transformed into a yellow color and lost its color pattern characteristics in its elytra (Figure 5D). These melanin-lacking ladybird beetles showed a normal activity state and mated normally. Thus, the fecundity of melanin-lacking and control ladybird beetles were calculated 10 days after eclosion. Our results show that there is no significant difference in fecundity and egg hatching rate between the ds*HaPn*r- and ds*GFP*-injected groups. The cumulative egg number within 15 days is 740 ± 99 and 781 ± 60.47 in the ds*GFP* and ds*HaPnr* group, respectively (Figure 5E). In addition, the hatching rate of eggs is 95.13 ± 0.94% and 90.87 ± 2.26% in the ds*GFP* and ds*HaPnr* group, respectively (Figure 5F).

### RNAi of *HaPnr-α* and *HaPnr-β* in the Fourth Instar Larvae and Its Effect on Elytral Melanization

To further understand the individual roles of the two splicing variant transcripts in the development and melanization of *H. axyridis*, we performed an experiment that injected *HaPnr-α and HaPnr-β* into the fourth instar larvae, respectively. The specific primer of dsRNA synthesis and RT-qPCR of each splicing variant was designed on a specific region (Supplementary Figure S3). Our results show that ds*HaPnr-α* dramatically reduced its transcript levels without significantly affecting the non-target mRNA levels on day 2 after injection (Figure 6A), but on day 4 after injection, the transcript levels of *HaPnr-β* was significantly increased as compared with control (Figure 6B). In addition, ds*HaPnr-β* dramatically reduced its transcript levels without significantly affecting the non-target mRNA levels on day 2 and 4 after injection (Figure 6C,D). The transcript levels of *HaPnr-α* and *HaPnr-β* were suppressed by 44% and 42% on day 2 after the injection of ds*HaPnr-α* and ds*HaPnr-β* as compared with their respective control (Figure 6A,C). Suppressing the expression level of *HaPnr-α* and *HaPnr-β* individually had no significant effect on the pupation and eclosion rate. The pupation rates were 83.3% and 85.7% on day 7 after injection with ds*HaPnr-α* and ds*HaPnr-β* as compared with their respective control, ds*GFP* injection (93.3%), respectively (Figure 7A,D). Furthermore, the eclosion rates were 85.33% and 82.4% on day 12 after injection with ds*HaPnr-α* and ds*HaPnr-β* as compared with their respective control, ds*GFP* injection (90.8%), respectively (Figure 7B,E). Suppressing the expression level of *HaPnr-α* in the fourth instar larvae resulted in decreased elytra melanin in both the *conspicua* and the *succinea* subgroup in *H. axyridis* (Figure 7C). In addition, suppressing the expression level of *HaPnr-β* in the fourth instar larvae resulted in decreased elytra melanin in the *succinea* subgroup only in *H. axyridis*. The *conspicua* subgroup of *H. axyridis* showed normal melanin levels when *HaPnr-β* was suppressed in the fourth instar larvae as compared with ds*GFP* injection (Figure 7F). However, we observed that the synthetic time of melanin was more than 12 h after eclosion, which was significantly longer than 2 h in the ds*GFP*-injected control.

## Discussion

The transcription factor *Pannier*, as a member of the GATA family, is involved in various biological processes during development in insects (Calleja et al., 2000; Buchberger et al., 2021). Recently, it was discovered to have a novel regulatory role in melanin synthesis in *H. axyridis* (Ando et al., 2018; Gautier et al., 2018; Ando and Niimi, 2019; Niimi and Ando, 2021). However, the detailed biological functions and regulated mechanisms of melanin in *H. axyridis* are still elusive. In the present study, we identified a *Pannier* homology from *H. axyridis* and characterized its cDNA and deduced amino acid sequences. Furthermore, we took advantage of the robust RNAi response of the insect to demonstrate its important biological function in pupation, eclosion and melanin synthesis in *H. axyridis.*

**FIGURE 2:**
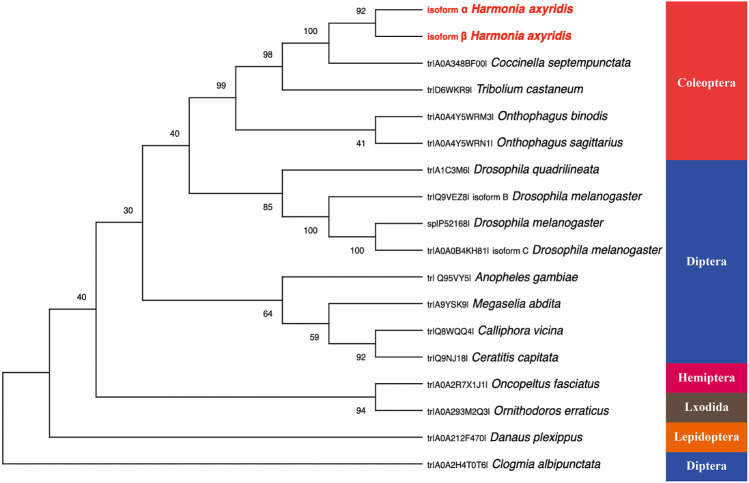
The rooted phylogenetic tree of deduced Pannier coding sequences from 15 insect species as constructed by the neighbor-jointing method. All the names used in the tree consist of the UniProt entry number and species name.

The model insect *D. melanogaster* study demonstrated that *Pannier* encodes two structurally related isoforms that are differentially expressed during development (Fromrntal-Ramain et al., 2008). Our results are consistent with the finding in *D. melanogaster* that *HaPnr* also has two splicing variants, *HaPnr-α* and *HaPnr-β* (Figure 1A). One of the most important features of the transcription factor is the zinc-finger domain (Han et al., 2012). In our study, the deduced protein sequence of the *HaPnr* gene exhibits the common features of the transcription factor: the *HaPnr-α* coded protein has two zinc finger domains, while the *HaPnr-β* coded protein has one zinc finger domain (Figure 1C), which is different from *D. melanogaster*, which has two zinc finger domains in the two isoforms, respectively (Fromrntal-Ramain et al., 2008; Valadez-Graham et al., 2012). Phylogenetic analysis of Pannier protein sequences of 15 insect species obtained from UniProt showed that the Pannier protein is conserved in the Coleoptera intragroup (Figure 2).

**FIGURE 3:**
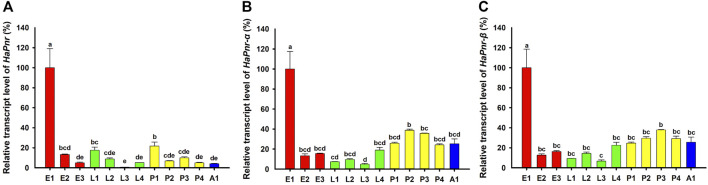
Relative transcript levels of *HaPnr* **(A)**, *HaPnr-α* **(B)**, and *HaPnr-β* **(C)** at different development stages of *H. axyridis* as determined by RT—qPCR. E1, E2, and E3 represent 1-, 2-, 3-day-old eggs; L1, L2, L3 and L4 represent first, second, third, and fourth instar larvae; P1, P2, P3 and P4 represent 1-, 2-, 3-, 4-day-old pupae; and A1 represents 1-day-old adults, respectively. Different letters above the standard error bars indicate significant differences based on ANOVA followed by Duncan’s multiple comparison test (*p* < 0.05). *H. axyridis* ribosomal protein 49 (*Harp49*) was used as an internal reference gene to normalize the differences among the samples. Relative expression levels for *HaPnr* were calculated based on the highest expressions of *HaPnr* in the one-day-old egg sample in developmental stage.

We found that *HaPnr*, including its splicing variants *HaPnr-α* and *HaPnr-β*, were constitutively expressed in all developmental stages. The highest expression of *HaPnr* occurred in the 1-day-old egg stage, and other expression peaks occurred in 1-day-old larvae and 1-day-old pupae (Figure 3A). Our results show that the *HaPnr* transcript peaked in early embryogenesis, then rapidly decreased during later embryogenesis, increased before the transition phase from embryo to the first instar larva and was maintained at an intermediate level during larva stage, then rapidly increased before the transition phase from larva to pupa stage and was also maintained at an intermediate level during the pupal stage. The high expression level of *HaPnr* in the initial stage of each metamorphosis process indicates that it plays a predominant role in the morphogenesis process in *H. axyridis*.

**FIGURE 4:**
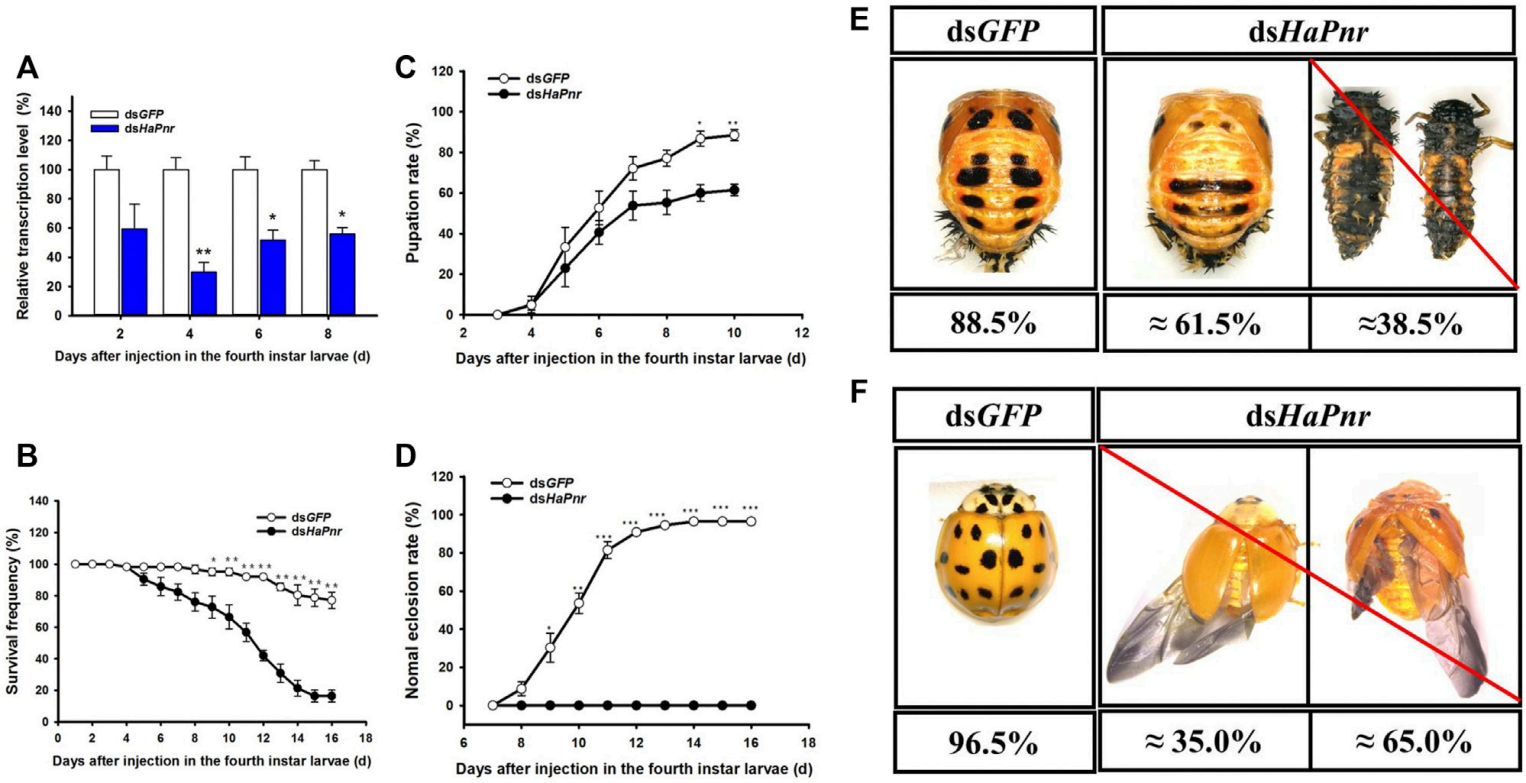
Suppression of *HaPnr* transcript in the fourth instar larvae of *H. axyridis* injected with *dsHaPnr* and *dsGFP* at 300 ng/larva as determined by RT-qPCR **(A)**. The time-dependent larval survival frequency **(B)**, pupation rate **(C)**, and normal eclosion rate **(D)** in *dsHaPnr* and *dsGFP*-treated larvae. The phenotype of pupae **(E)** and adults **(F)** that injected with *dsGFP* and *dsHaPnr* with 300 ng/larva in the fourth instar larvae. The results are presented as the mean and standard errors of three replicates; each was performed with an RNA sample prepared from three insects. Asterisks above the standard error bars indicate significantly differences (Duncan tests, *p* < 0.05).

To determine the detailed biological function of *HaPnr* during metamorphosis (pupation and eclosion), we performed a functional assay to suppress the expression level of *HaPnr* in the fourth instar larvae. Specifically, in the fourth instar larvae, injection of ds*HaPnr* correnponding to a common region of the *HaPnr-α* and *HaPnr-β* transcript resulted in the significant decrease of its transcript level (Figure 4A). The results indicated that *HaPnr* plays an important role in the development process. Such suppression resulted in high mortality within 16 days after the injection (Figure 4B). The results of *HaPnr* was suppressed in the fourth instar larvae resulted in two major lines of evidence to support its essential role in pupation and eclosion process. First, suppressing the expression level of *HaPnr* led to a significantly reduced pupation rate (Figure 4C), and the un-pupated larvae eventually died after 1 week (Figure 4E). Second, even when a small proportion of the ds*HaPnr*-injected larvae were able to pupate, these pupae were unable to emerge into normal adults, and these abnormal adults died within 10 days after eclosion (Figure 4D,F). Our results are in agreement with a previous study in *D. melanogaster* demonstrating that the first exon of *Pannier* is inserted into a transposable element carrying the *Gal4* gene, resulting in higher mortality in homozygote *Pannier-Gal4* flies (Heitzler et al., 1996; Valadez-Graham et al., 2012).

**FIGURE 5:**
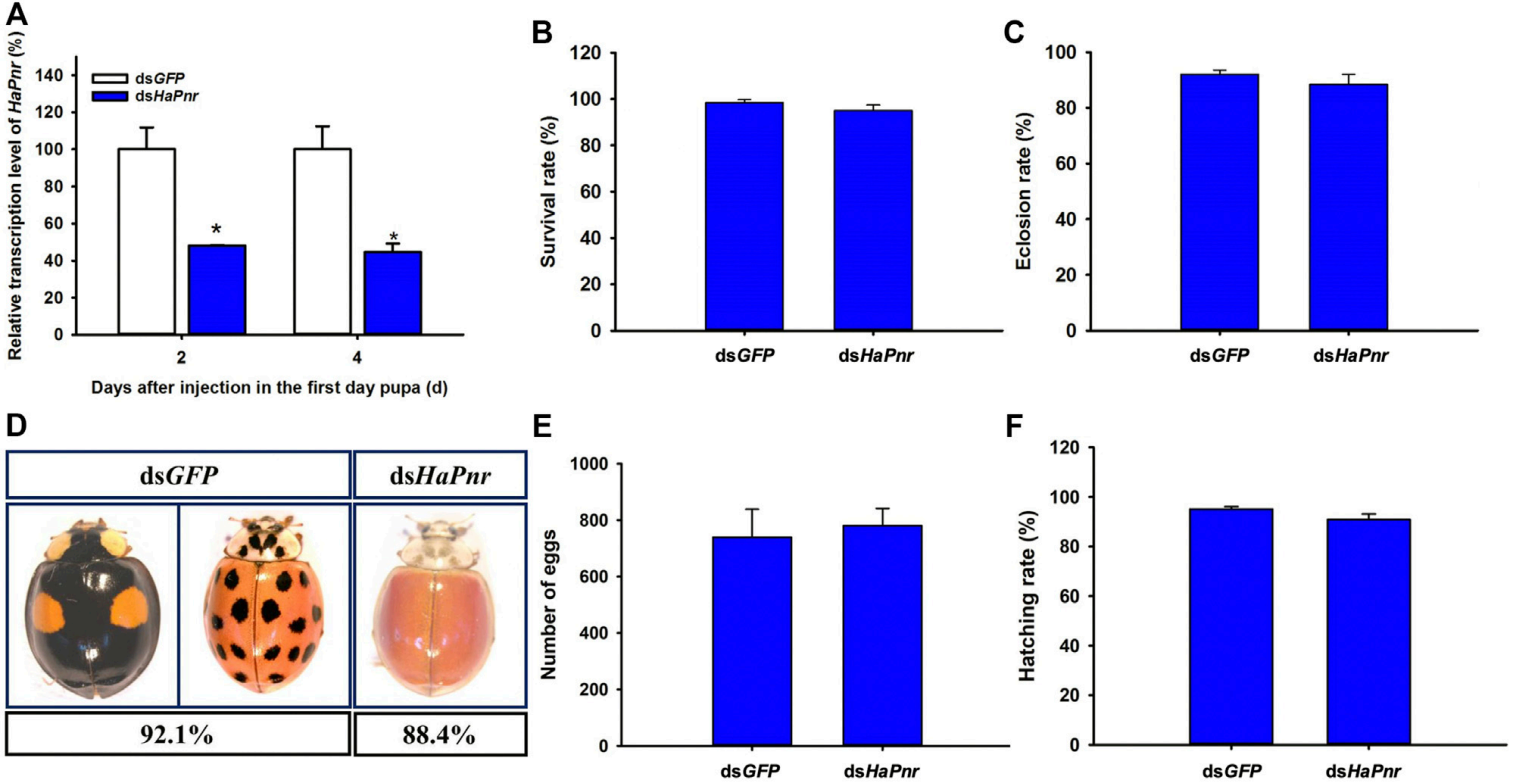
Suppression of *HaPnr* transcript in the one-day-old pupa of *H. axyridis* injected with *dsHaPnr* and *dsGFP* at 300 ng/pupa as determined by RT-qPCR **(A)**. The survival rate **(B)** and eclosion rate **(C)** of the one-day-old pupae injected with *dsHaPnr* and *dsGFP*, respectively. The phenotype of adults after the one-day-old pupae were injected with *dsHaPnr* and *dsGFP*, respectively **(D)**. The cumulative egg number of three pairs of ladybirds within 15 days **(E)** and the hatching rate of eggs **(F)**. The results are presented as the mean and standard errors of three replicates; each was performed with an RNA sample prepared from three insects. Asterisks above the standard error bars indicate significantly different (Duncan tests, *p* < 0.05).

To further confirm the role of *HaPnr* in the metamorphosis process, we continue to perform detailed functional analyses by using RNAi to suppress the expression level of *HaPnr* in one-day-old pupae. Specifically, the injection of ds*HaPnr* corresponding to a common region of its two splicing variants transcript resulted in a dramatic suppression of its transcript level in one-day-old pupae (Figure 5A). However, suppression of *HaPnr* expression in the early pupa stage did not affect the eclosion process (Figure 5C). ds*HaPnr*-injected pupae were able to pupate into adults, but with an abnormal phenotype in which melanin disappeared from the head, pronotum and elytra (Figure 5D). We found that ladybird beetles missing melanin could mate normally and perform oviposition. Additionally, there were no significant differences in fecundity and egg hatching ratio between the ds*HaPnr*- and ds*GFP*-injection groups (Figure 5E,F). Previous research reported that *Pannier* could regulate imaginal disc development in insects (Maurel-Zaffran and Treisman, 2000; Orose et al., 2010; Buchberger et al., 2021). Imaginal discs are defined as clusters of undifferentiated embryonic cells in holometabolous insects that proliferate during larval stages, then differentiate during the pupal stage (Blair, 2009). In the present study, severe lethality occurred when the expression level of *HaPnr* was suppressed in the fourth instar larvae; however, no mortality was observed when early pupae (1 day old) were injected with ds*HaPnr*. Through comprehensive analysis of the difference in lethality resulting from *HaPnr* suppression in the fourth instar larvae and early pupa (1 day old) stage, our results demonstrate that the crucial regulation time of *HaPnr* in development is before the pupa stage (the fourth instar larvae or pre-pupae stage). Furthermore, we speculate that *HaPnr* may regulate development of the wing disc in the final larval stage due to the malformed hind wing which was obtained in the absence of *HaPnr* expression (Figure 4F).

Beyond the regulatory role of *HaPnr* in pupation and eclosion process, it also plays a predominant role in regulating melanin synthesis in *H. axyridis*. The suppression of *HaPnr* in the fourth instar larvae resulted in melanin disorder in pupae and melanin disappearing from the head, pronotum and elytra in adults (Figure 4E,F). Furthermore, melanin-lacking *H. axyridis* were also obtained when *HaPnr* was suppressed in one-day-old pupae (Figure 5D). Our results are consistent with recent studies focusing on color pattern polymorphism research, which also demonstrated that *HaPnr* could regulate melanin synthesis in *H. axyridis* (Gautier et al., 2018; Ando et al., 2018). In our previous study, suppression of the expression level of dopa decarboxylase (*DDC*) also resulted in a melanin-absent phenotype, which is the crucial gene in dopamine melanin synthesis. Even though the melanin disappeared in the elytra in *H. axyridis*, we can easily discern the characteristic of color pattern because of the original melanin area transforming into a light-yellow color when *HaDDC* was suppressed (Xiao et al., 2020; Chen et al., 2019b). However, we did not discern the color pattern in *H. axyridis* when *HaPnr* was suppressed in the fourth instar larvae or one-day-old pupae due to the whole elytra represent the same yellow color (Figures 4F, 5D). We speculated that *HaPnr* has additional functions which could regulate pigment cell differentiation. In a previous study, the researchers also presented a similar viewpoint that *Pannier* may be expressed in specific cells in the forewing in *H. axyridis,* which promotes differentiation in black pigment cells and suppresses differentiation into red pigment cell (Niimi and Ando, 2021).

**FIGURE 6:**
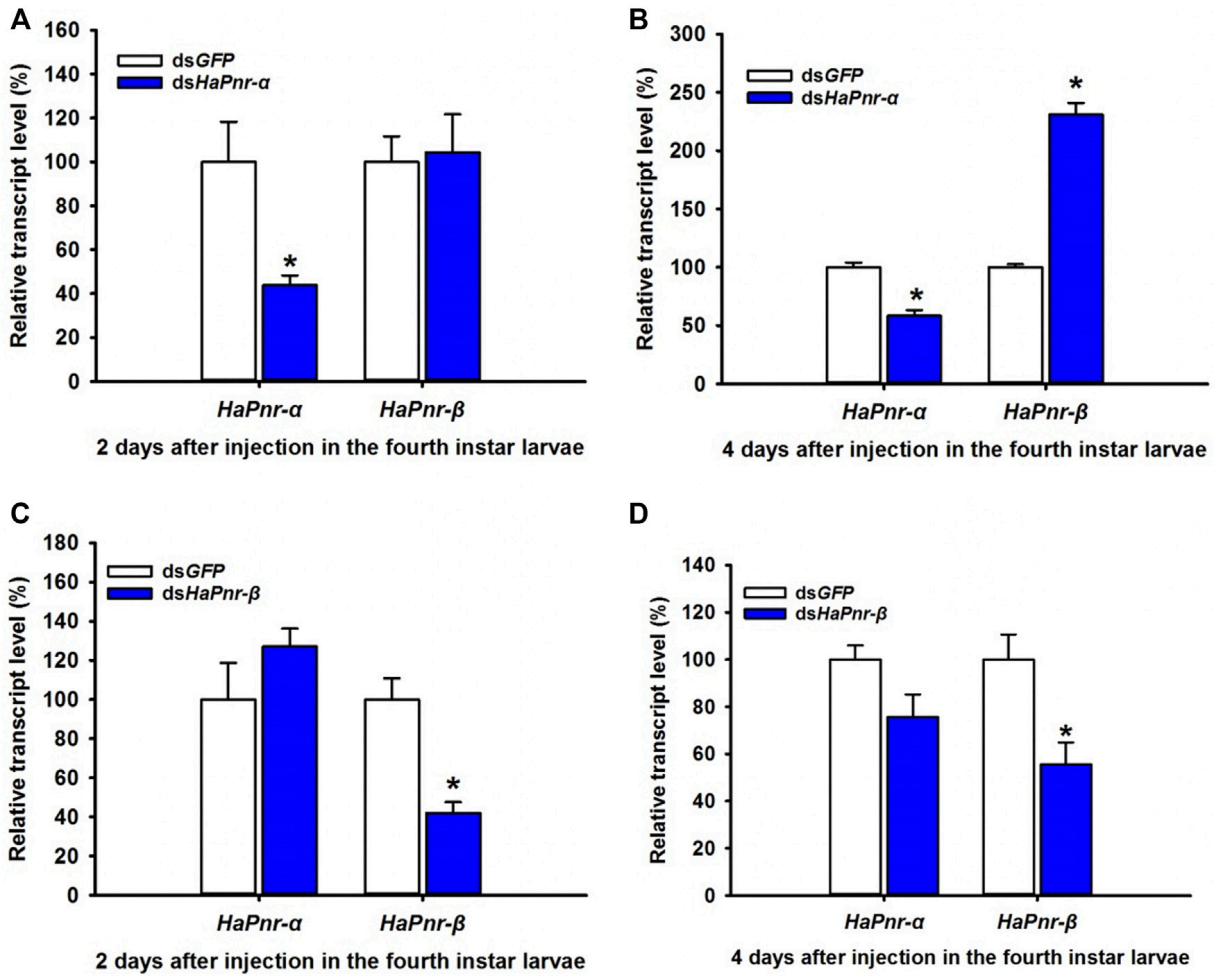
Suppression of *HaPnr-α* **(A,B)** and *HaPnr-β* **(C,D)** transcript in the fourth instar larvae of *H. axyridis* injected with dsHaPnr-α, dsHaPnr-β, and dsGFP at 300 ng/larva as determined by RT-qPCR.

**FIGURE 7:**
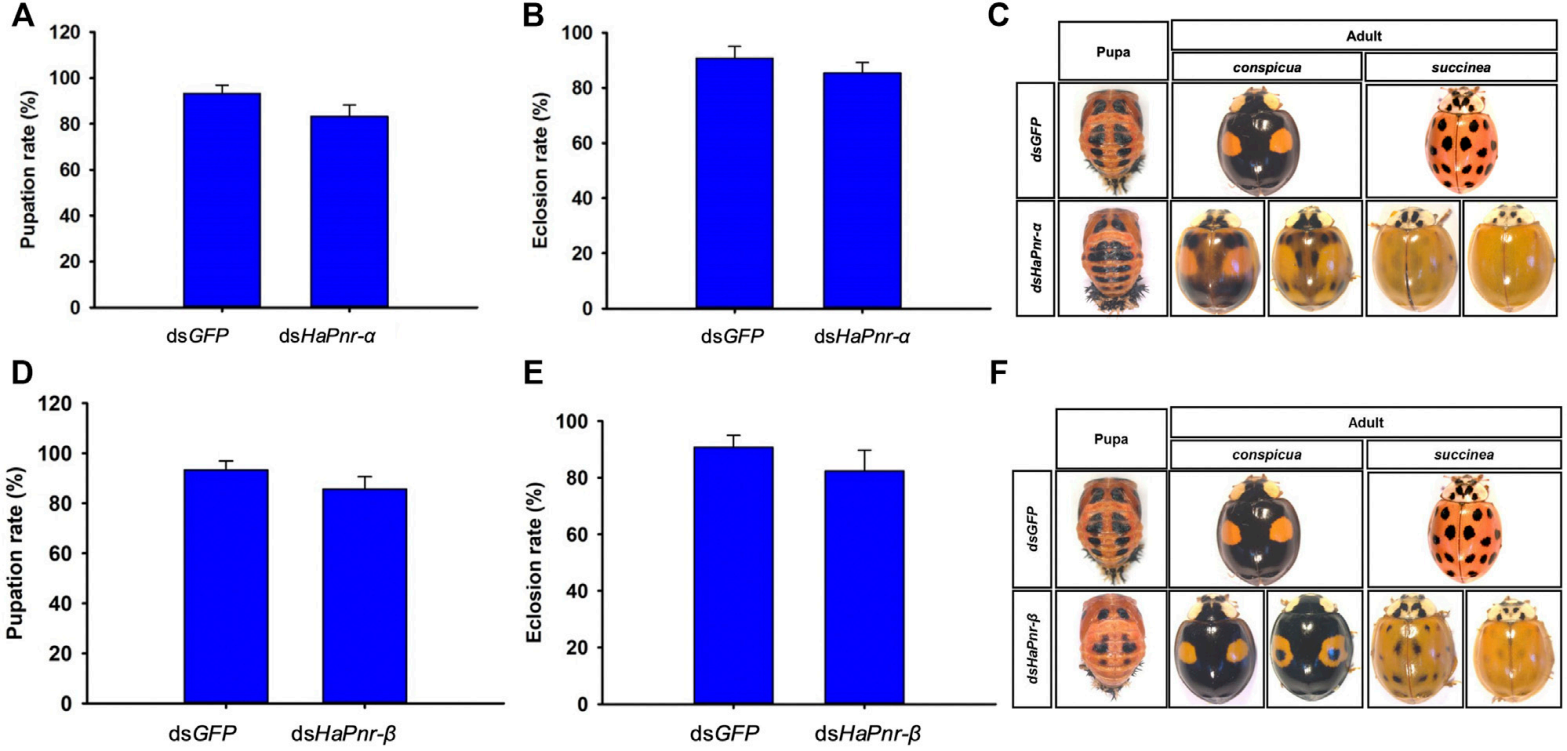
The pupation rate in ds*HaPnr-α* **(A)** and ds*HaPnr-β* **(D)** injected groups compared with ds*GFP*-treated larvae, respectively. The eclosion rate in ds*HaPnr-α* **(B)** and ds*HaPnr-β* **(E)** injected group compared with ds*GFP*-treated larvae, respectively. The pupae and adult’s phenotype after the fourth instar larvae injected with ds*HaPnr-α* **(C)** and ds*HaPnr-β* **(F)**. The results are presented as the mean and standard errors of three replicates; each was performed with an RNA sample prepared from three insects. Asterisks above the standard error bars indicate significantly different (Duncan tests, *p* < 0.05).

Indeed, the mode of action of *Pannier* splicing variants was reported in *D. melanogaster*, displaying antagonistic activities in the regulation of *wingless* expression during imaginal disc development (Fromrntal-Ramain et al., 2008). In order to analysis the sole role of *HaPnr* splicing variants in pupation and eclosion process in *H. axyridis*, special dsRNA corresponding to the specific region of *HaPnr-α* and *HaPnr-β* was designed and injected into the fourth instar larvae in *H. axyridis* (Supplementary Figure S3). Specifically, the injection of dsRNA corresponding to a specific region of *HaPnr-α* and *HaPnr-β* transcript resulted in a dramatic suppression of their transcript level in the fourth instar larvae, respectively (Figure 6A–D). Unlike with the suppression of the expression level of *HaPnr* in the fourth instar larvae, suppressing the expression level of its splicing variant individually has no significant effect on pupation and eclosion (Figure 7A,B,D,E), indicating that any of the individual splice variants are able to perform the *HaPnr* genetic functions in development. We continue to analyze the phenotype induced by *HaPnr-α* or *HaPnr-β* being suppressed in the fourth instar larvae individually. Our results show that the melanin was significantly decreased in the *succinea* subgroup in *H. axyridis* when both *HaPnr-α* and *HaPnr-β* were suppressed in the fourth instar larvae, individually. Even though the melanin was synthesized normally in the *conspicua* pattern in *H. axyridis*, the coloration time was significantly longer as compared with the control (Figure 7C,F). As compared with the phenotype when *HaPnr* was suppressed in the fourth instar larvae and one-day-old pupae, melanin was totally absent, and the color pattern characteristic was lost. Our present data highlight that *HaPnr* function in regulated melanin synthesis is achieved by two structurally related isoforms. In addition, through comprehensive analysis of the distribution of zinc-finger domain in *HaPnr-α* and *HaPnr-β*, we speculate that *HaPnr-α* may play a predominant role in regulating melanin synthesis with two zinc finger domain and *HaPnr-β* may play an auxiliary role in regulating melanin synthesis with one zinc finger domain.

In summary, previous research revealed evolutionally conserved mechanisms of *Pannier* in regulating imaginal disc development, dorsal closure, and the formation of ommateum and bristle. However, much of the evidence for such behaviors in insects comes from studies of the *Pannier* mutants of *D. melanogaster*. Thus, our results provide, for the first time, crucial evidence that *HaPnr* plays an essential role in insect metamorphosis in *H. axyridis* by using RNAi. In addition, *HaPnr* also regulates melanin synthesis under the combined action of its two splicing variants, *HaPnr-α* and *HaPnr-β*.

## Data Availability

The original contributions presented in the study are publicly available. This data can be found here: https://www.ncbi.nlm.nih.gov/, GenBank: ON186775, ON186776.
