# Supplementary material for: The Molecular Properties and Roles of Pannier in Harmonia axyridis’s Metamorphosis and Melanin Synthesis
Source: Front Physiol. 2022 May 3;13:909258. doi: 10.3389/fphys.2022.909258 (PMC9110671; doi:10.3389/fphys.2022.909258)
Supplement: Supplementary file 1 [file Image1.pdf]

Supplementary Material

```
1    ATG TTC CAC ACC GGC GCC GGC GGC AAC GGC TAT GGC GAC GGC AAC GCG GGT TTC CAC CAA CAC CTT CAG CAG TCC CCC GTC TAC GTG CCG
1     M   F   H   T   G   A   G   G   N   G   Y   G   D   G   N   A   G   F   H   Q   H   L   Q   Q   S   P   V   Y   V   P

91   AGC AGC AGG GCC GTA CCG CAC CAA TAT TCC CCG GCG GCA GGC ACC CAT TTC GGC GCC GCC GCC CAC CAG GGA GGC TGG GCT CAC GCC GGC
31    S   S   R   A   V   P   H   Q   Y   S   P   A   A   G   T   H   F   G   A   A   A   H   Q   G   G   W   A   H   A   G

181  GGT TCC TAC GGC GAC ATG GCC TCG CAG GCG CAC GGT CTT GGG GGC GCC GCT CAC GCC TCT CCC CTC TCC GCC GGT CAG TTT TAC ACC CAG
61    G   S   Y   G   D   M   A   S   Q   A   H   G   L   G   G   A   A   H   A   S   P   L   S   A   G   Q   F   Y   T   Q

271  AAC ATG GTC ATG TCC TCC TGG CGG GCC TAC GAC GGC TCT GGA TTC CAG CGG ACG TCA CCT TAT GAG AGT GCC ATG GAG TTC CAG TTC GGA
91    N   M   V   M   S   S   W   R   A   Y   D   G   S   G   F   Q   R   T   S   P   Y   E   S   A   M   E   F   Q   F   G

361  GAG GGC AGG GAG TGC GTC AAT TGT GGG GCC ATT TCC ACC CCT CTG TGG AGG AGA GAT GGT ACA GGG CAC TAC TTG TGT AAC GCT TGT GGT
121   E   G   R   E   C   V   N   C   G   A   I   S   T   P   L   W   R   R   D   G   T   G   H   Y   L   C   N   A   C   G

451  TTG TAC CAC AAG ATG AAT GGG ATG AAC AGA CCC TTA ATC AAA CCA TCC AAA AGA CTG ACC GCA ACC AGA CGC TTG GGA CTG TGC TGC ACG
151   L   Y   H   K   M   N   G   M   N   R   P   L   I   K   P   S   K   R   L   T   A   T   R   R   L   G   L   C   C   T

541  AAC TGC GGC ACC AGG ACG ACG ACG CTG TGG CGT CGT AAC AAC GAC GGC GAA CCG GTG TGC AAC GCC TGT GGC CTA TAT TTC AAG TTG CAC
181   N   C   G   T   R   T   T   T   L   W   R   R   N   N   D   G   E   P   V   C   N   A   C   G   L   Y   F   K   L   H

631  GGA GTG AAT AGG CCC CTG GCC ATG CGC AAG GAC GGA ATC CAG ACC CGT AAG AGG AAG CCG AAA AAA CAA GGT GGT GGT AGT GGG GAG CGA
211   G   V   N   R   P   L   A   M   R   K   D   G   I   Q   T   R   K   R   K   P   K   K   Q   G   G   G   S   G   E   R

721  GAC GAC AGT AGC TCC ACC TCC GTA GAA GAC GGC AAG ACA CCA GTA AGC ATA TCA AAC CAA CAG CAA CAC TCA AAC CAA CAA GTA CCT TCG
241   D   D   S   S   S   T   S   V   E   D   G   K   T   P   V   S   I   S   N   Q   Q   Q   H   S   N   Q   Q   V   P   S

811  TCA TCT CAA AAC CAT CCC AAT CAT CAA CAT TCA CCA GAT AAC AAA GTT ACC CAG ACC TTG GAA CGA CCT TAT CTA AGT CCA GCG TCA CTG
271   S   S   Q   N   H   P   N   H   Q   H   S   P   D   N   K   V   T   Q   T   L   E   R   P   Y   L   S   P   A   S   L

901  TTA CCA TCT TCA TCG AGC CTG GTG AAG AGC GAA CCG GGA TAC GAT TAC AGC TGC CTG CAG AAC CAA GGA TAC CCT TAC CAG CAA ATC TTC
301   L   P   S   S   S   S   L   V   K   S   E   P   G   Y   D   Y   S   C   L   Q   N   Q   G   Y   P   Y   Q   Q   I   F

991  GGG TTC CCA GGT GCT GGA CCG ACA AAC CCA GAA TTA GCG TAT CAT CAC CAA CAT CAC GTA ACA GCT TCT GCC AAA CTG ATG GCT ACG ACA
331   G   F   P   G   A   G   P   T   N   P   E   L   A   Y   H   H   Q   H   H   V   T   A   S   A   K   L   M   A   T   T

1081 TAA
361   *
```

Supplementary Figure 1. The CDS region and deduced amino acid sequences of *Pannier* gene from *H. axyridis* (*HaPnr-α*). The amino acid sequence was numbered from the start of its predicted mature protein. The start codon ATG was bolded and underlined, and the stop codon TAA at the end of the coding region was bolded and marked with an asterisk. The second exon, alternative splicing part, was showed red fonts. The blue boxes represent one predicted GATA-type domain, and the orange boxes represent the other one of HaPnr-α.

```
1    ATG TTC CAC ACC GGC GCC GGC GGC AAC GGC TAC GGC GAC GGC AAC GCG GGT TTC CAC CAA CAC CTT CAG CAG TCC CCC GTC TAC GTG CCG
1     M   F   H   T   G   A   G   G   N   G   Y   G   D   G   N   A   G   F   H   Q   H   L   Q   Q   S   P   V   Y   V   P

91   AGC AGC AGG GCC GTA CCG CAC CAA TAT TCC CCG GCG GCA GGC ACC CAT TTC GGC GCC GCC GCC CAC CAG GGA GGC TGG GCT CAC GCC GGC
31    S   S   R   A   V   P   H   Q   Y   S   P   A   A   G   T   H   F   G   A   A   A   H   Q   G   G   W   A   H   A   G

181  GGT TCC TAC GGC GAC ATG GCC TCG CAG GCG CAC GGT CTT GGG GGC GCC GCT CAC GCC TCT CCC CTC TCC GCC GGT CAG TTC TAC ACC CAG
61    G   S   Y   G   D   M   A   S   Q   A   H   G   L   G   G   A   A   H   A   S   P   L   S   A   G   Q   F   Y   T   Q

271  AAC ATG GTC ATG TCC TCC TGG CGG GCC TAC GAC GGC TCT GGA TTC CAA CGG ACG TCA CCT TAT GAA CAT GGA TAC GAA AGT AAC CCG TAC
91    N   M   V   M   S   S   W   R   A   Y   D   G   S   G   F   Q   R   T   S   P   Y   E   H   G   Y   E   S   N   P   Y

361  CCC AAC CAC CGG CAA CCC TAC TGG AAT CTA GCG CCA TAT CGA AAA CCG TCA AAA ATA CTA ACC GCA ACC AGA CGC TTG GGA CTG TGC TGC
121   P   N   H   R   Q   P   Y   W   N   L   A   P   Y   R   K   P   S   K   I   L   T   A   T   R   R   L   G   L   C   C

451  ACG AAC TGC GGC ACC AGG ACG ACG ACG CTG TGG CGT CGT AAC AAC GAC GGC GAA CCG GTG TGC AAC GCC TGT GGC CTA TAT TTC AAG TTG
151   T   N   C   G   T   R   T   T   T   L   W   R   R   N   N   D   G   E   P   V   C   N   A   C   G   L   Y   F   K   L

541  CAC GGA GTG AAT AGG CCC CTG GCC ATG CGC AAG GAC GGC ATC CAG ACC CGT AAG AGG AAG CCG AAA AAA CAA GGT GGT GGT AGT GGG GAG
181   H   G   V   N   R   P   L   A   M   R   K   D   G   I   Q   T   R   K   R   K   P   K   K   Q   G   G   G   S   G   E

631  CGA GAC GAC AGT AGC TCC ACC TCC GTA GAA GAC GGT AAG ACA CCA GTA AGC ATA TCA AAC CAA CAG CAA CAC TCA AAC CAA CAA GTA CCT
211   R   D   D   S   S   S   T   S   V   E   D   G   K   T   P   V   S   I   S   N   Q   Q   Q   H   S   N   Q   Q   V   P

721  TCG TCA TCT CAA AAC CAT CCC AAT CAT CAA CAT TCA CCA GAT AAC AAA GTT ACC CAG ACC TTG GAA CGA CCT TAT CTA AGT CCA GCG TCA
241   S   S   S   Q   N   H   P   N   H   Q   H   S   P   D   N   K   V   T   Q   T   L   E   R   P   Y   L   S   P   A   S

811  CTG TTA CCA TCT TCA TCG AGC CTG GTG AAG AGC GAA CCG GGA TAC GAT TAC AGC TGC CTG CAG AAC CAA GGA TAC CCT TAC CAG CAA ATC
271   L   L   P   S   S   S   S   L   V   K   S   E   P   G   Y   D   Y   S   C   L   Q   N   Q   G   Y   P   Y   Q   Q   I

901  TTC GGG TTC CCA GGT GCT GGA CCG ACA AAC CCA GAA TTA GCG TAT CAT CAC CAA CAT CAC GTA ACA GCT TCT GCC AAA CTG ATG GCT ACG
301   F   G   F   P   G   A   G   P   T   N   P   E   L   A   Y   H   H   Q   H   H   V   T   A   S   A   K   L   M   A   T

991  ACA TAA
331   T   *
```

Supplementary Figure 2. The CDS region and deduced amino acid sequences of *Pannier* gene from *H. axyridis* (*HaPnr-β*). The amino acid sequence was numbered from the start of its predicted mature protein. The start codon ATG was bolded and underlined, and the stop codon TAA at the end of the coding region was bolded and marked with an asterisk. The second exon, alternative splicing part, was showed red fonts. The orange boxes represent the only one predicted GATA-type domain of HaPnr-β.

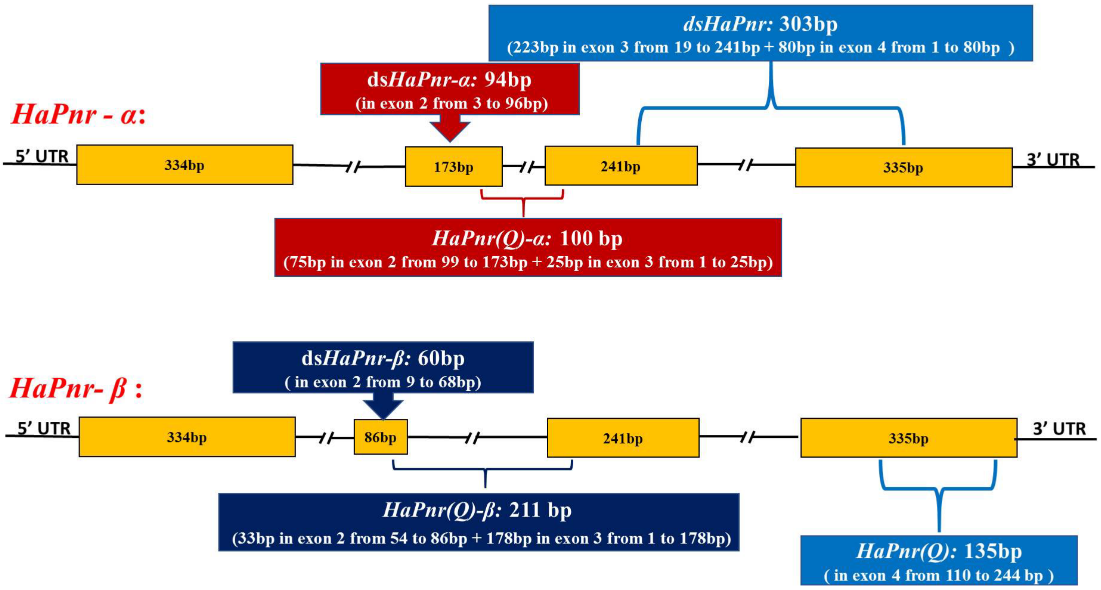

Supplementary Figure 3. Schematic diagram of the primer of dsRNA and RT-qPCR designed for *HaPnr*, *HaPnr-α* and *HaPnr-β*.
